# New York Pathological Society, January 27th, 1847

**Published:** 1847-03

**Authors:** 


New York Pathological Society, January 27th, 1847.

Dr. Wood exhibited a *polypus uteri* which he had removed by ligature from a woman in whom it had existed for nine years. It was about the size of a Vergaloo pear, and hung by a well defined pedicle from the fundus. Several fibrous tumours were attached to other parts of the uterus, appreciable by the hand externally applied, varying in size from that of a marble to that of the fist. In three days after the application of the ligature, the polypus came away without hæmorrhage; it had lost nearly half of its bulk. Confirmatory of his previously expressed opinion, Dr. W. had found this polypus, as he had others, supplied by one large artery in the pedicle; this gave off other small ones. It was difficult to secure arteries after their section in all fibrous tissues, as room, owing to their elasticity, is not given for the hold of the ligature beyond the tenaculum. He had attempted it, uncuccessfully, upon this specimen. Hence the great danger of *excising* these growths, which he considered a bad and hazardous practice. He exhibited an instrument well adapted to the purpose of ligating polypi, and, coinciding with Dr. Montgomery in the belief of the identity of the three forms of uterine fibrous tumours, ând his condemnation of the knife, strongly recommended the perusal of his late valuable paper on the subject of this disease. Dr. W. stated further, that he had recently confined a woman, whose child (a female) when five weeks old, weighed two and a quarter pounds. It was born in the seventh month, and for two weeks did not nurse. It is now doing well.

Dr. Van Buren exhibited the parts taken from a patient with *aneurism of the femoral artery, and communication of the artery and vein.* The gentleman was twenty-one; he had been wounded two and a half years ago in an affray, with a pistol ball, which entered below the ant. sup. spinous process of the ilium, traversed the front of the thigh, and came out on the inside of the symphysis, under the skin. At the moment, much hæmorrhage ensued, arrested by pressure. He recovered entirely in six weeks; on getting about, he discovered a pulsating tumour in the middle of the thigh, three inches below Poupart's ligament. When examined, on his arrival here, it was irregular and ill-defined, three or four inches in circumference, and over it, *a very unusually distinct thrill* could be felt. The tumour was in contact with Poupart's ligament. The external iliac artery was tied by Professor Mott on the 16th of December, and on the 22d he died, gangrene having extended over the whole limb, commencing at the foot. The operation was ill borne; severe pain was felt at the moment of tightening the ligature for several minutes, followed by numbness of the whole limb; soon after he had pain and oppression at the heart. Next day there was much agitation, with oppression about the chest and pain in the belly, and the temperature of the limb was eight or nine degrees lower than that of the opposite side. The pulse was never as full as before, after the operation.

*Post mortem* (which was difficult on account of the state of the parts.) A little lymph upon the peritoneum around the wound, not affecting that of the adjacent intestines. The ligature was applied about one and a quarter inches above the circumflexa and epigastric arteries, and a clot of about an inch in length above it. The artery began to be atheromatous just below Poupart's ligament. A true aneurism communicated with the artery just above thé origin of the arteria profunda, a little larger in size than a partridge egg, and of a regular oval shape like a pouch, having a small jug-like neck, and containing coagula formed before the ligature of the artery. It was enveloped by gristly cellular tissue. Immediately under the sac, and blended with it, was the anterior crural nerve, much larger than natural in appearance, from thickening, probably, of its cellular neurilemma.

The vein along side was healthy, but thickened, and the cellular tissue at this point, infiltrated and indurated. *Just here existed a communication between the vein and artery.* A little cyst of bony shell was attached to the surface of the vein, just where the saphena dips into it.

The case Dr. V. B. believed to be unique; *an aneurismal varix, and a true aneurism* of the femoral artery. The large quantity of indurated cellular tissue above the disease, and the exaggerated thrill gave the idea of a much larger aneurism than really existed. The injury done to the artery had caused disease of its coats and aneurism. There was also decided hypertrophy of the heart, with induration of its tissue; no ossific deposits. Each cavity contained a polypus, quite white and apparently formed before death. Dr. V. B. said that a patient operated on by Mr. Ramsden, with subclavian aneurism, went on well for some days, with slight oppression of the heart, and died suddenly. A polypus existed in the descending vena cava, depending into the right auricle, which acted as a valve and caused death. Hence, he inferred, that there might be something in the ligating of large arteries, which led to this condition. The heart, in Dr. M.'s patient, beat very irregularly after the operation and intermitted.—*N. Y. Annalist.*

